# Differential metabonomic profiles of primary hepatocellular carcinoma tumors from alcoholic liver disease, HBV-infected, and HCV-infected cirrhotic patients

**DOI:** 10.18632/oncotarget.18397

**Published:** 2017-06-07

**Authors:** Ding Cao, Can Cai, Mingxin Ye, Junhua Gong, Menghao Wang, Jinzheng Li, Jianping Gong

**Affiliations:** ^1^ Department of Hepatobiliary Surgery, The Second Affiliated Hospital, Chongqing Medical University, Chongqing, 400010, China; ^2^ Department of Gastroenterology, The Second Affiliated Hospital of Chongqing Medical University, Chongqing, 400010, China

**Keywords:** hepatocellular carcinoma, alcoholic liver disease, hepatitis B virus, cirrhosis, metabonomic

## Abstract

Our objective was to comparatively profile the metabolite composition of primary hepatocellular carcinoma (HCC) tumors from alcoholic liver disease (ALD), hepatitis B virus (HBV)-infected, and hepatitis C virus (HCV)-infected cirrhotic patients. Primary HCC tumors were collected from ALD, HBV-infected, and HCV-infected cirrhotic patients (n=20 each). High-resolution magic-angle spinning proton nuclear magnetic resonance spectroscopy and metabonomic data analysis were performed to compare HCC tumors from the three groups. Sensitivity analyses were performed to determine the effects of diabetes, high body mass index, and smoking status. Metabonomic pathway analyses were conducted to identify dysregulated pathways. Three metabolites were significantly differentiated between ALD and HBV-infected patients, which were distinguishable by changes in ketone body, glycerolipid, and phenylalanine metabolism. Five metabolites were significantly differentiated between ALD and HCV-infected patients, which were distinguishable by changes in ketone body, alanine/aspartate/glutamate, and phenylalanine metabolism. Six metabolites were significantly differentiated between HBV-infected and HCV-infected patients, which were distinguishable by changes in ketone body, tyrosine, and alanine/aspartate/glutamate metabolism. In conclusion, this is the first study to demonstrate that the metabolic phenotypes of primary HCC tumors vary significantly across ALD, HBV-infected, and HCV-infected cirrhotic patients.

## INTRODUCTION

Hepatocellular carcinoma (HCC) remains a key research area due to HCC’s high mortality rates and complicated pathogenesis [1]. Although previous genetic studies have identified frequently mutated genes in HCC [2, 3], HCC tumorigenesis is not simply determined by innate genetic differences but by a myriad of environmental factors [4, 5]. For example, almost 80% of HCC cases are due to underlying chronic hepatitis B (HBV) and hepatitis C (HCV) infection with estimated relative risks of 15-20× and 2×, respectively, as compared to non-infected individuals [6]. Moreover, alcohol abuse contributes to HCC pathogenesis, with HCC risk increasing linearly above daily alcohol consumption levels of 60 g (i.e., six drinks or shots) [6]. Although chronic HBV infection, chronic HCV infection, and alcohol abuse have all been acknowledged to contribute to HCC development, the mechanisms underlying the pathogenesis of HCC in each of these three clinical scenarios remains unclear.

The introduction of metabonomics -- the quan-titative analysis of the metabolic response of a biological system to external stimuli [7] -- has provided more information on HCC by focusing on the metabolite end-products that are affected by environmental factors [8]. Current metabonomic studies on HCC have mainly focused on identifying characteristic metabolites in the serum or urine to identify potential biomarkers for future clinical applications [9]. Although blood-based and urine-based metabonomic analyses are easy to perform and can reveal important peripheral pathological changes [9], metabonomic analysis of the actual liver tissue sampled by biopsy or surgery can be useful in identifying the underlying pathogenic changes in HCC tumors in response to external stimuli.

To this end, several recent studies have applied various metabonomic approaches to examine the differences between HCC tumor tissue and adjacent benign liver tissue [10–12]. However, no metabonomic study to date has yet comparatively profiled the metabolite composition of primary HCC tumors from alcoholic liver disease (ALD), HBV-infected, and HCV-infected cirrhotic patients. Therefore, the objective of this study was to comparatively profile the metabolite composition of primary HCC tumors from ALD, HBV-infected, and HCV-infected cirrhotic patients using high-resolution magic-angle spinning proton nuclear magnetic resonance (HRMAS ^1^H-NMR) spectroscopy. Analysis of the metabolic differences between these three types of HCC tumors should provide important insights into any differences in their pathogenesis. These insights can provide guidance for future basic research as well as applied research on diagnostics and therapeutics for HCC.

## RESULTS

The key clinical and demographic characteristics of the included patients are detailed in Table 1. The three experimental groups were statistically similar across all characteristics with the notable exception of alcohol consumption in the ALD group (*P*<0.05).

**Table 1:** Key Clinical and Demographic Characteristics of the Included Patients

Parameter	ALD	HBV	HCV
N	20	20	20
Median age (yrs) (range)	56 (32-87)	56 (32-82)	55 (31-83)
Sex (M/F %)	80%/20%	70%/30%	80%/20%
Diabetes (%)	25%	35%	30%
Median BMI (range)	22.1 (18.4-28.0)	21.0 (17.3-26.2)	22.4 (17.8-26.6)
Smoker (%)	40%	50%	40%
Median alcohol consumption (g/week) (range)	1160* (0-1760)^†^	210 (0-270)	250 (0-290)
Median ALT (IU/L) (range)	79.0 (50.0-128.2)	76.44 (58.6-100.4)	77.55 (5.90-100.8)
Median AST (IU/L) (range)	55.3 (34.1-87.2)	74.63 (59.1-95.3)	75.17 (59.0-94.5)
Median alkaline phosphatase (IU/L) (range)	346.1 (261.8-462.4)	279.5 (244.0-320.6)	280.16 (244.2-320.7)
Median total bilirubin (mg/dL) (range)	1.05 (0.69-1.62)	1.19 (0.95-1.47)	1.18 (0.95-1.45)
Median direct bilirubin (mg/dL) (range)	0.12 (0.05-0.29)	0.15 (0.10-0.23)	0.15 (0.10-0.23)
Median albumin (g/dL) (range)	3.46 (3.15-3.80)	3.63 (3.47-3.85)	3.67 (3.46-3.80)
Median ECOG score (0-4) (range)	1 (0-4)	1 (0-4)	1 (0-4)
Median CLIP score (range)	1 (0-3)	1 (0-3)	1 (0-3)
Median Child-Pugh score (A-C) (range)	A (A-C)	A (A-C)	A (A-C)
Median tumor size (cm) (range)	4.1 (1.4-16.5)	4.0 (1.3-15.5)	3.7 (1.3-16.3)
Median tumor grade (G1-G4) (range)	G2 (G1-G3)	G2 (G1-G3)	G2 (G1-G3)

*P<0.05

^†^Zero alcohol consumption is included in the range of the ALD group, as some ALD participants had quit drinking alcohol.

### Primary metabonomic analyses

**Figure 1:**
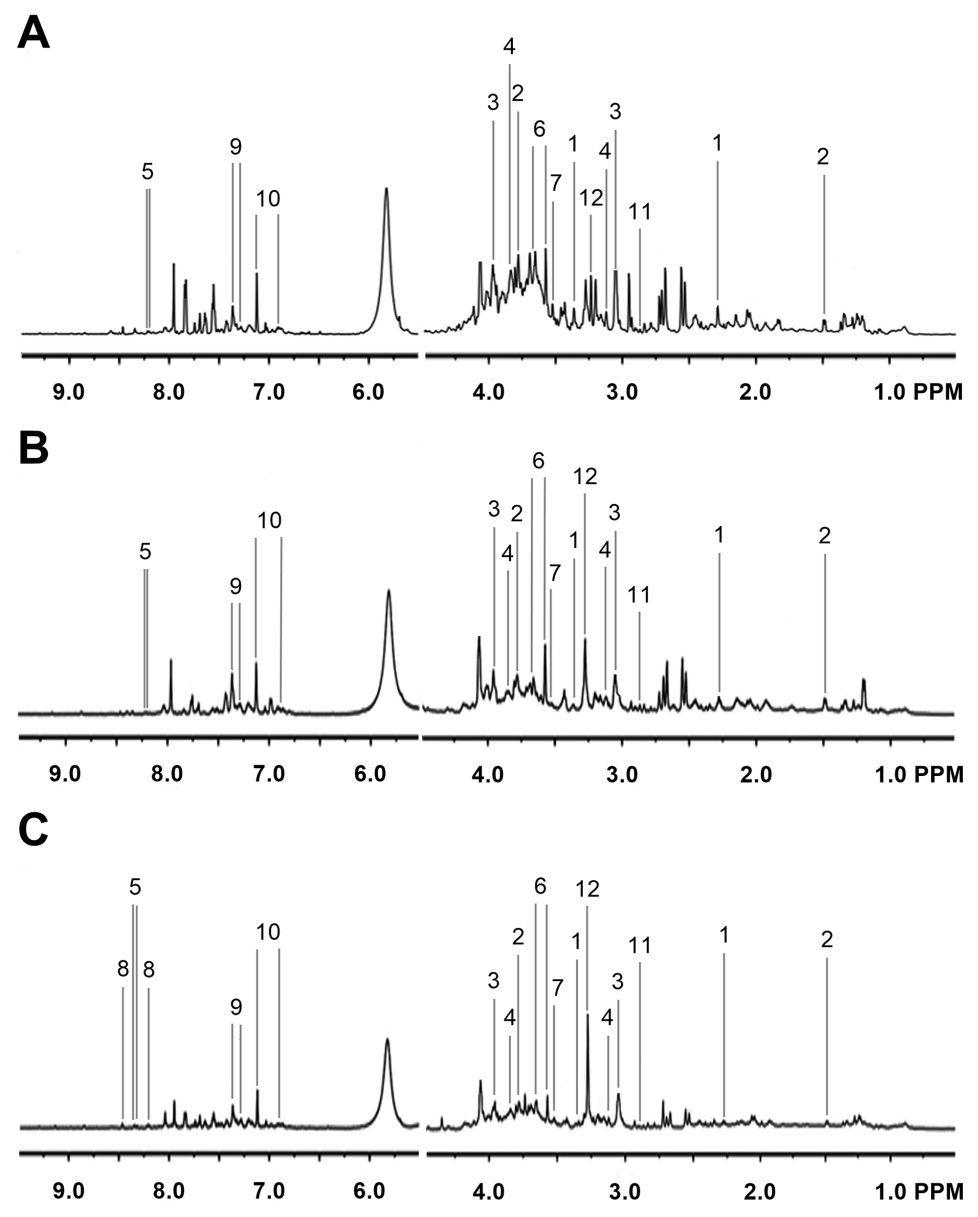
Representative ^1^H NMR Spectra from Primary HCC Tumors Representative ^1^H NMR spectra of primary HCC tumors extracted from **(A)** ALD, **(B)** HBV-infected, and **(C)** HCV-infected patients. Labeled metabolites: (1) acetoacetate, (2) alanine, (3) creatine, (4) ethanolamine, (5) hypoxanthine, (6) glycerol, (7) glycine, (8) NADP, (9) phenylacetate, (10) *p*-hydroxyphenylacetic acid, (11) trimethylamine, and (12) trimethylamine N-oxide. The left panels have been vertically re-scaled to match the right panels, and the vertical scale of the whole spectra has been increased by a factor of 2× to make the peaks clearer.

**Figure 2:**
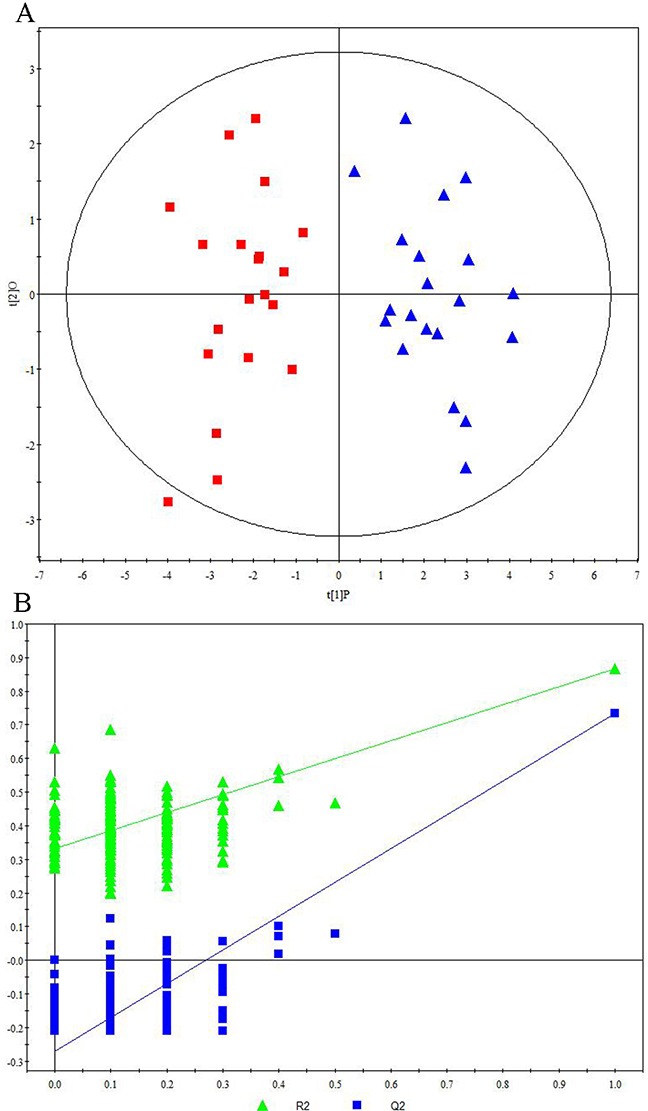
Metabonomic Analysis Differentiating Primary HCC Tumors from ALD Patients and HBV-Infected Patients **(A)** OPLS-DA score plots showing a clear discrimination between primary HCC tumors from ALD patients (red squares) and HBV-infected patients (blue circles). **(B)** 200× permutation testing showing the original R^2^ and Q^2^ values (top right; R^2^ =0.982, Q^2^=0.857) as significantly higher than corresponding permuted values (top right), demonstrating the OPLS-DA model’s robustness.

**Figure 3:**
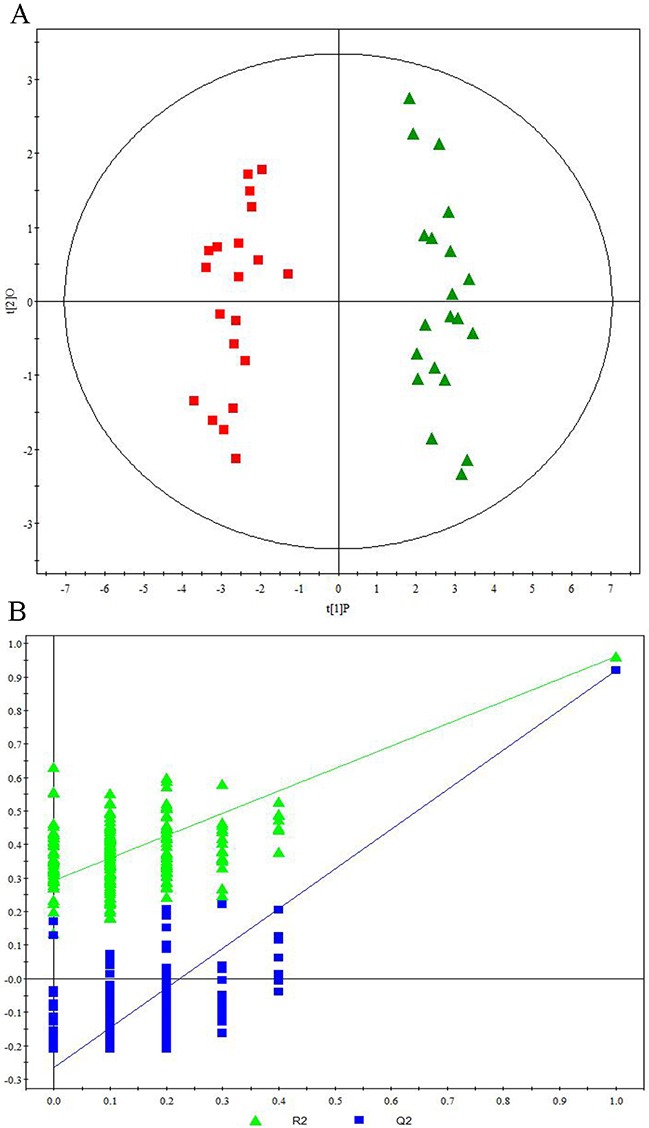
Metabonomic Analysis Differentiating Primary HCC Tumors from ALD Patients and HCV-Infected Patients **(A)** OPLS-DA score plots showing a clear discrimination between primary HCC tumors from ALD patients (red squares) and HCV-infected patients (green diamonds). **(B)** 200× permutation testing showing the original R^2^ and Q^2^ values (top right; R^2^ =0.963, Q^2^=0.925) as significantly higher than corresponding permuted values (top right), demonstrating the OPLS-DA model’s robustness.

**Figure 4:**
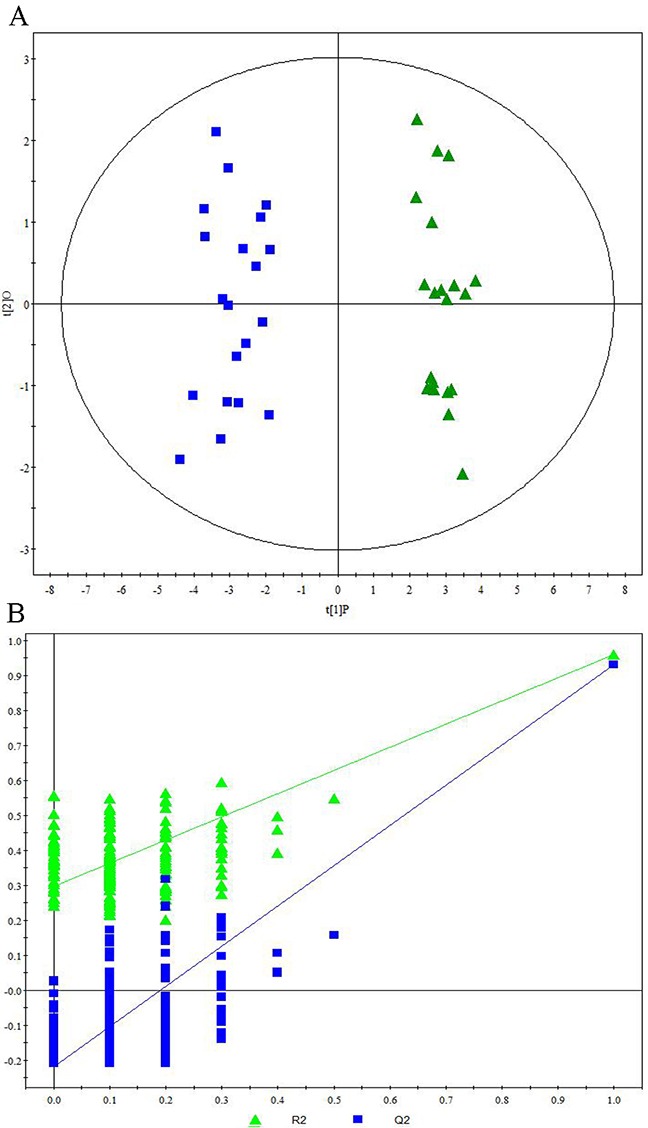
Metabonomic Analysis Differentiating Primary HCC Tumors from HBV-Infected Patients and HCV-Infected Patients **(A)** OPLS-DA score plots showing a clear discrimination between primary HCC tumors from HBV-infected patients (blue circles) and HCV-infected patients (green diamonds). **(B)** 200× permutation testing showing the original R^2^ and Q^2^ values (top right; R^2^ =0.960, Q^2^=0.936) as significantly higher than corresponding permuted values (top right), demonstrating the OPLS-DA model’s robustness.

Representative ^1^H NMR spectra of primary HCC tumors from ALD (Figure 1A), HBV-infected (Figure 1B), and HCV-infected cirrhotic patients (Figure 1C) are provided. We first performed three comparative metabonomic analyses: (i) ALD versus HBV-infected cirrhotic patients, (ii) ALD versus HCV-infected cirrhotic patients, and (iii) HBV-infected versus HCV-infected cirrhotic patients. The non-supervised principal component analysis (PCA) plots from these three metabonomic analyses are provided in Supplementary Figure 1. The OPLS-DA score plots (with one predictive and one orthogonal component) from the supervised analysis showed a clear discrimination between the metabolic profiles of (i) ALD versus HBV-infected cirrhotic patients (Figure 2A), (ii) ALD versus HCV-infected cirrhotic patients (Figure 3A), and (iii) HBV-infected versus HCV-infected cirrhotic patients (Figure 4A). Permutation testing demonstrated the OPLS-DA model’s robustness for all three comparisons (Figure 2B, 3B, and 4B). Tables listing the statistically significant metabolites differentiating (i) ALD versus HBV-infected cirrhotic patients (Supplementary Table 1), (ii) ALD versus HCV-infected cirrhotic patients (Supplementary Table 2), and (iii) HBV-infected versus HCV-infected cirrhotic patients (Supplementary Table 3) have been provided.

### Sensitivity analyses

To examine the effects of diabetes, high BMI, and smoking status, we conducted sensitivity analyses through excluding diabetics (Supplementary Figure 2), overweight individuals (Supplementary Figure 3), and smokers (Supplementary Figure 4). The resulting OPLS-DA score plots still showed a clear discrimination between (i) ALD versus HBV-infected cirrhotic patients (Supplementary Figures 2A, 3A, and 4A), (ii) ALD versus HCV-infected cirrhotic patients (Supplementary Figures 2B, 3B, and 4B), and (iii) HBV-infected versus HCV-infected cirrhotic patients (Supplementary Figures 2C, 3C, and 4C). Tables listing the statistically significant metabolites after excluding diabetics (Supplementary Tables 4-6), overweight individuals (Supplementary Tables 7-9), and smokers (Supplementary Tables 10-12) have been provided.

### ALD versus HBV comparison

Through comparing the differential metabolites across the parent analysis and all three sensitivity analyses, we determined that three metabolites persisted across all ALD vs. HBV sensitivity analyses: acetoacetate (↓HBV), glycerol (↓HBV), and *p*-hydroxyphenylacetate (↓HBV). These findings suggest that these metabolites can differentiate primary HCC tumors from ALD and HBV-infected cirrhotic patients irrespective of diabetes, high BMI, or smoking status (Supplementary Figure 5). From our metabonomic pathway analysis, primary HCC tumors from ALD and HBV-infected cirrhotic patients were significantly distinguishable by changes in three metabolic pathways: synthesis and degradation of ketone bodies (impact=0.70, *P*=2.41E-08), glycerolipid metabolism (impact=0.19, *P*=1.79E-07), and tyrosine metabolism (impact=0.06, *P*=7.09E-09) (Table 2, Supplementary Figure 5).

**Table 2:** Differential Metabonomic Pathways for ALD versus HBV-Infected HCC Tumors by Impact Ranking

Pre-sensitivity analysis	*P*-value	-Log(*P*)	Holm *P*	FDR	Impact
Synthesis and degradation of ketone bodies	2.41E-08	17.54	4.58E-07	9.65E-08	0.70
Glycerolipid metabolism	1.79E-07	15.54	2.69E-06	5.12E-07	0.19
Glycine, serine and threonine metabolism	3.74E-04	7.89	4.23E-03	3.74E-04	0.19
Tyrosine metabolism	7.09E-09	18.76	1.42E-07	9.65E-08	0.06
Glycerophospholipid metabolism	4.68E-07	14.58	6.08E-06	1.17E-06	0.06
Butanoate metabolism	2.41E-08	17.54	4.58E-07	9.65E-08	0.04
Propanoate metabolism	2.41E-08	17.54	4.58E-07	9.65E-08	0.03
Primary bile acid biosynthesis	3.74E-04	7.89	4.23E-03	3.74E-04	0.01
Valine, leucine and isoleucine degradation	2.41E-08	17.54	4.58E-07	9.65E-08	0.00
Galactose metabolism	1.79E-07	15.54	2.69E-06	5.12E-07	0.00
Phenylalanine metabolism	3.53E-04	7.95	4.23E-03	3.74E-04	0.00
Purine metabolism	3.74E-04	7.89	4.23E-03	3.74E-04	0.00
Lysine degradation	3.74E-04	7.89	4.23E-03	3.74E-04	0.00
Cyanoamino acid metabolism	3.74E-04	7.89	4.23E-03	3.74E-04	0.00
Glutathione metabolism	3.74E-04	7.89	4.23E-03	3.74E-04	0.00
Methane metabolism	3.74E-04	7.89	4.23E-03	3.74E-04	0.00
Thiamine metabolism	3.74E-04	7.89	4.23E-03	3.74E-04	0.00
Porphyrin and chlorophyll metabolism	3.74E-04	7.89	4.23E-03	3.74E-04	0.00
Nitrogen metabolism	3.74E-04	7.89	4.23E-03	3.74E-04	0.00
Aminoacyl-tRNA biosynthesis	3.74E-04	7.89	4.23E-03	3.74E-04	0.00
**Post-sensitivity analysis**	***P*-value**	**-Log(*P***)	**Holm *P***	**FDR**	**Impact**
Synthesis and degradation of ketone bodies	2.41E-08	17.54	1.69E-07	3.86E-08	0.70
Glycerolipid metabolism	1.79E-07	15.54	5.37E-07	2.05E-07	0.19
Tyrosine metabolism	7.09E-09	18.76	5.67E-08	3.86E-08	0.06
Butanoate metabolism	2.41E-08	17.54	1.69E-07	3.86E-08	0.04
Propanoate metabolism	2.41E-08	17.54	1.69E-07	3.86E-08	0.03
Valine, leucine and isoleucine degradation	2.41E-08	17.54	1.69E-07	3.86E-08	0.00
Galactose metabolism	1.79E-07	15.54	5.37E-07	2.05E-07	0.00
Phenylalanine metabolism	3.53E-04	7.95	3.53E-04	3.53E-04	0.00

### ALD versus HCV comparison

We determined that five metabolites –– ace-toacetate (↓HCV), alanine (↓HCV), creatine (↓HCV), phenylacetate (↓HCV), and trimethylamine N-oxide (↑HCV) -- persisted across all ALD vs. HCV sensitivity analyses. These findings suggest that these metabolites can differentiate between primary HCC tumors from ALD and HCV-infected cirrhotic patients irrespective of diabetes, high BMI, or smoking status (Supplementary Figure 6). From our metabonomic pathway analysis, primary HCC tumors from ALD and HCV-infected cirrhotic patients were significantly distinguishable by changes in three metabolic pathways: synthesis and degradation of ketone bodies (impact=0.70,*P*=1.95E-15), alanine, aspartate and glutamate metabolism (impact=0.06, *P*=2.47E-11), and phenylalanine metabolism (impact=0.05, *P*=5.18E-13) (Table 3, Supplementary Figure 6).

**Table 3:** Differential Metabonomic Pathways for ALD versus HCV-Infected HCC Tumors by Impact Ranking

Pre-sensitivity analysis	*P*-value	-Log(*P*)	Holm *P*	FDR	Impact
Synthesis and degradation of ketone bodies	1.95E-15	33.87	4.29E-14	8.57E-15	0.70
Glycine, serine and threonine metabolism	5.33E-13	28.26	8.81E-12	1.68E-12	0.19
Alanine, aspartate and glutamate metabolism	2.47E-11	24.43	3.70E-10	4.93E-11	0.06
Phenylalanine metabolism	5.18E-13	28.29	8.81E-12	1.68E-12	0.05
Butanoate metabolism	1.95E-15	33.87	4.29E-14	8.57E-15	0.04
Taurine and hypotaurine metabolism	2.47E-11	24.43	3.70E-10	4.93E-11	0.03
Arginine and proline metabolism	3.92E-11	23.96	4.32E-10	7.19E-11	0.03
Propanoate metabolism	1.95E-15	33.87	4.29E-14	8.57E-15	0.03
Primary bile acid biosynthesis	1.84E-08	17.81	1.47E-07	1.93E-08	0.01
Purine metabolism	2.34E-10	22.18	2.34E-09	3.96E-10	0.01
Valine, leucine and isoleucine degradation	1.95E-15	33.87	4.29E-14	8.57E-15	0.00
Tyrosine metabolism	1.95E-15	33.87	4.29E-14	8.57E-15	0.00
Cysteine and methionine metabolism	2.47E-11	24.43	3.70E-10	4.93E-11	0.00
Selenoamino acid metabolism	2.47E-11	24.43	3.70E-10	4.93E-11	0.00
Aminoacyl-tRNA biosynthesis	1.19E-09	20.55	1.07E-08	1.87E-09	0.00
Lysine degradation	1.84E-08	17.81	1.47E-07	1.93E-08	0.00
Cyanoamino acid metabolism	1.84E-08	17.81	1.47E-07	1.93E-08	0.00
Glutathione metabolism	1.84E-08	17.81	1.47E-07	1.93E-08	0.00
Thiamine metabolism	1.84E-08	17.81	1.47E-07	1.93E-08	0.00
Porphyrin and chlorophyll metabolism	1.84E-08	17.81	1.47E-07	1.93E-08	0.00
Nitrogen metabolism	1.84E-08	17.81	1.47E-07	1.93E-08	0.00
Methane metabolism	6.78E-04	7.30	6.78E-04	6.78E-04	0.00
**Post-sensitivity analysis**	***P*-value**	**-Log(*P***)	**Holm *P***	**FDR**	**Impact**
Synthesis and degradation of ketone bodies	1.95E-15	33.87	2.73E-14	5.46E-15	0.70
Alanine, aspartate and glutamate metabolism	2.47E-11	24.43	1.97E-10	3.14E-11	0.06
Phenylalanine metabolism	5.18E-13	28.29	4.66E-12	1.21E-12	0.05
Butanoate metabolism	1.95E-15	33.87	2.73E-14	5.46E-15	0.04
Taurine and hypotaurine metabolism	2.47E-11	24.43	1.97E-10	3.14E-11	0.03
Arginine and proline metabolism	3.92E-11	23.96	1.97E-10	4.23E-11	0.03
Propanoate metabolism	1.95E-15	33.87	2.73E-14	5.46E-15	0.03
Glycine, serine and threonine metabolism	3.92E-11	23.96	1.97E-10	4.23E-11	0.00
Valine, leucine and isoleucine degradation	1.95E-15	33.87	2.73E-14	5.46E-15	0.00
Tyrosine metabolism	1.95E-15	33.87	2.73E-14	5.46E-15	0.00
Cysteine and methionine metabolism	2.47E-11	24.43	1.97E-10	3.14E-11	0.00
Selenoamino acid metabolism	2.47E-11	24.43	1.97E-10	3.14E-11	0.00
Aminoacyl-tRNA biosynthesis	2.47E-11	24.43	1.97E-10	3.14E-11	0.00
Methane metabolism	9.15E-04	7.00	9.15E-04	9.15E-04	0.00

### HBV versus HCV comparison

We determined that six metabolites –– acetoacetate (↓HCV), alanine (↓HCV),*p*-hydroxyphenylacetate (↑HCV), hypoxanthine (↓HCV), NADP (↑HCV), and trimethylamine N-oxide (↑HCV) -- persisted across all HBV vs. HCV sensitivity analyses. These findings suggest that these metabolites can differentiate between primary HCC tumors from HBV-infected and HCV-infected cirrhotic patients irrespective of diabetes, high BMI, or smoking status (Supplementary Figure 7). From our metabonomic pathway analysis, primary HCC tumors from HBV-infected and HCV-infected cirrhotic patients were significantly distinguishable by changes in three metabolic pathways: synthesis and degradation of ketone bodies (impact=0.70,*P*=1.59E-09), tyrosine metabolism (impact=0.06, *P*=3.93E-11), and alanine, aspartate and glutamate metabolism (impact=0.06, *P*=1.63E-12) (Table 4, Supplementary Figure 7).

**Table 4:** Differential Metabonomic Pathways for HBV-Infected versus HCV-Infected HCC Tumors by Impact Ranking

Pre-sensitivity analysis	*P*-value	-Log(*P*)	Holm *P*	FDR	Impact
Synthesis and degradation of ketone bodies	1.59E-09	20.26	1.59E-08	2.46E-09	0.70
Tyrosine metabolism	3.93E-11	23.96	4.32E-10	9.54E-11	0.06
Alanine, aspartate and glutamate metabolism	1.63E-12	27.14	2.61E-11	4.62E-12	0.06
Phenylalanine metabolism	1.09E-12	27.55	1.85E-11	4.62E-12	0.05
Butanoate metabolism	1.59E-09	20.26	1.59E-08	2.46E-09	0.04
Taurine and hypotaurine metabolism	1.63E-12	27.14	2.61E-11	4.62E-12	0.03
Arginine and proline metabolism	1.00E-07	16.12	6.01E-07	1.31E-07	0.03
Propanoate metabolism	1.59E-09	20.26	1.59E-08	2.46E-09	0.03
Glutathione metabolism	6.77E-03	4.99	1.36E-02	6.77E-03	0.01
Purine metabolism	1.28E-07	15.87	6.01E-07	1.56E-07	0.01
Glycine, serine and threonine metabolism	1.00E-07	16.12	6.01E-07	1.31E-07	0.00
Cysteine and methionine metabolism	1.63E-12	27.14	2.61E-11	4.62E-12	0.00
Selenoamino acid metabolism	1.63E-12	27.14	2.61E-11	4.62E-12	0.00
Aminoacyl-tRNA biosynthesis	1.63E-12	27.14	2.61E-11	4.62E-12	0.00
Valine, leucine and isoleucine degradation	1.59E-09	20.26	1.59E-08	2.46E-09	0.00
Methane metabolism	1.06E-03	6.85	3.17E-03	1.20E-03	0.00
Nicotinate and nicotinamide metabolism	6.77E-03	4.99	1.36E-02	6.77E-03	0.00
**Post-sensitivity analysis**	***P*-value**	**-Log(*P***)	**Holm *P***	**FDR**	**Impact**
Synthesis and degradation of ketone bodies	1.59E-09	20.26	1.43E-08	2.39E-09	0.70
Tyrosine metabolism	3.93E-11	23.96	3.93E-10	9.82E-11	0.06
Alanine, aspartate and glutamate metabolism	1.63E-12	27.14	2.45E-11	4.89E-12	0.06
Butanoate metabolism	1.59E-09	20.26	1.43E-08	2.39E-09	0.04
Taurine and hypotaurine metabolism	1.63E-12	27.14	2.45E-11	4.89E-12	0.03
Propanoate metabolism	1.59E-09	20.26	1.43E-08	2.39E-09	0.03
Glutathione metabolism	6.77E-03	4.99	1.36E-02	6.77E-03	0.01
Purine metabolism	1.28E-07	15.87	6.41E-07	1.75E-07	0.01
Cysteine and methionine metabolism	1.63E-12	27.14	2.45E-11	4.89E-12	0.00
Selenoamino acid metabolism	1.63E-12	27.14	2.45E-11	4.89E-12	0.00
Aminoacyl-tRNA biosynthesis	1.63E-12	27.14	2.45E-11	4.89E-12	0.00
Valine, leucine and isoleucine degradation	1.59E-09	20.26	1.43E-08	2.39E-09	0.00
Phenylalanine metabolism	1.99E-04	8.52	7.96E-04	2.49E-04	0.00
Methane metabolism	1.06E-03	6.85	3.17E-03	1.22E-03	0.00
Nicotinate and nicotinamide metabolism	6.77E-03	4.99	1.36E-02	6.77E-03	0.00

## DISCUSSION

In this study, we comparatively profiled the metabolite composition of primary HCC tumors from ALD, HBV-infected, and HCV-infected cirrhotic patients using a HRMAS ^1^H-NMR spectroscopic approach. We found that the key differential metabolites from these three primary HCC tumor types were significantly different, even after controlling for key risk factors (i.e., diabetes, high BMI, and smoking status). Through metabonomic pathway analyses, these three primary HCC tumor types displayed significant differences in their affected metabolic pathways, even after controlling for the foregoing risk factors. Although previous metabolomic studies on HCC tumors have demonstrated upregulation of glycolysis, gluconeogenesis, and β-oxidation coupled with TCA cycle downregulation [10, 14], this is the first study to demonstrate that the metabolic phenotypes of primary HCC tumors vary significantly across ALD, HBV-infected, and HCV-infected cirrhotic patients.

Notably, the three types of HCC tumors were significantly distinguishable by changes in ketone body metabolism with the following associated metabolite profiles: acetoacetate (ALD>HBV>HCV), alanine (ALD and HBV>HCV), and glycerol (ALD>HBV and HCV). Previous plasma metabonomic research has demonstrated that ketone body and ketogenic amino acid levels are significantly increased in cirrhotic patients, suggesting that peripheral ketone body utilization is impaired in cirrhotic patients [15]. As acetoacetate is the primary ketogenic product [15], our findings further suggest that ketogenesis is the most pronounced in ALD HCC tumors, followed by HBV-infected HCC tumors, and lastly HCV-infected HCC tumors in cirrhotic patients. Notably, this hypothesis is further supported by the observed higher glycerol levels in ALD relative to HBV-infected and HCV-infected HCC tumors, suggesting increased triglyceride catabolism and fatty acid oxidation contributing to enhanced ketone body synthesis in ALD HCC tumors in cirrhotic patients [15]. Interestingly, previous research has shown an inverse relationship between blood ketone body levels and alanine levels on account of alanine’s inhibition of ketogenesis [16]. Here, we found a positive relationship between intracellular (as opposed to blood) ketone body levels and alanine levels, suggesting that primary HCC tumors in cirrhotic patients may import alanine as a feedback mechanism to regulate enhanced ketone body generation. This finding is consistent with previous research showing higher alanine levels in primary HCC tumors relative to recurrent HCC tumors [17].

In addition, the three types of HCC tumors were significantly distinguishable by changes in glycerolipid metabolism characterized by differential glycerol levels (ALD>HBV and HCV). A chromatography-time of flight/mass spectrometry (GC-TOF/MS)/Random Forests metabonomic analysis by Gao et al. investigating the serum metabolic changes along the disease progression from HBV infection-to-liver cirrhosis-to-HCC found downregulated glycerol levels and related perturbation in glycerolipid metabolism across all three disease stages [18]. Previous work has suggested that the reduced glycerol levels commonly observed in HCC tumor cells may be related to downregulated expression of the aquaglyceroporin AQP9, which serves as a channel for glycerol and water transport [19, 20]. On this basis, our findings suggest there may be higher AQP9 expression in primary HCC tumors arising from ALD relative to primary HCC tumors arising from HBV and HCV infection in cirrhotic patients.

Additionally, the three types of HCC tumors were significantly distinguishable by changes in phenylalanine/tyrosine metabolism characterized by differential *p*-hydroxyphenylacetate levels (HCV>ALD>HBV) and differential phenylacetate levels (ALD and HBV>HCV). In healthy human liver cells, phenylalanine degradation proceeds by the standard hepatic pathway: phenylalanine → tyrosine → 4-hydroxyphenylpyruvic acid → homogentisic acid → CO_2_ [21]. However, in the presence of cirrhosis, the standard hepatic pathway is inhibited; as a result, phenylalanine is catabolized to *p*-hydroxyphenylacetate via the following alternative decarboxylation pathway: phenylalanine → tyrosine → tyramine → *p*-hydroxyphenylacetate[21]. Our current findings suggest that the three types of HCC tumors may display differential activity levels in this alternative decarboxylation pathway with HCV-infected HCC tumors showing the highest activity, followed by ALD HCC tumors, followed by HBV-infected HCC tumors. In addition, the initial conversion step of phenylalanine-to-tyrosine is catalyzed by the enzyme phenylalanine hydroxylase (PH) [21]. Previous metabolomic research has demonstrated that HCC tumors display a decreased phenylalanine-to-tyrosine ratio, suggesting PH inhibition in HCC tumors [10]. When PH is inhibited, phenylalanine degradation proceeds via the following minor pathway: phenylalanine → phenylpyruvate → phenylacetate [22]. Therefore, our current findings suggest more pronounced inhibition of PH in ALD and HBV-infected HCC tumors relative to HCV-infected HCC tumors in cirrhotic patients.

The foregoing findings provide insights into the differential pathogenesis of HCC under the three clinical conditions (i.e., ALD, HBV-infected, and HCV-infected) that may be clinically relevant. For example, here we demonstrated that ketogenesis in cirrhotic HCC patients was most pronounced in ALD HCC tumors, followed by HBV-infected HCC tumors, and lastly HCV-infected HCC tumors. As ketogenic hepatocytes display distinct molecular changes, such as enhanced ROS production as well as upregulated extracellular signal-regulated kinase 1/2 (Erk1/2) and p38 mitogen-activated protein kinase (MAPK) phosphorylation via a PKC- and Ras-based mechanism [23], our results suggest that tailored chemotherapeutics which selectively target these dysregulated pathways may show improved efficacy in ALD HCC patients. Therefore, further investigation on the differentially dysregulated pathways in ALD, HBV-infected, and HCV-infected HCC tumors should provide valuable insights for tailored chemotherapeutic approaches.

There are several limitations to this study. First, all HCC tumors examined here were derived from a population of Han Chinese patients. Therefore, ethnic biases may have adversely affected the findings. Future metabonomic studies on this topic should aim to recruit ethnically heterogeneous populations from several international study sites to control for any potential biases. Second, all HCC tumors examined here were derived from patients that had not received radiotherapy, chemotherapy, or antiviral therapy. Therefore, we were unable to analyze the effects of these interventions upon the metabolic profiles of each experimental group. Third, only one metabonomic platform – ^1^H NMR HRMAS -- was applied in the present study. This approach fundamentally limits the coverage of metabolites, so future metabonomic studies on this topic should use multiple platforms to broaden metabolite coverage. Fourth, although our metabonomic analysis controlled for several HCC risk factors (i.e., diabetes, high BMI, and smoking status) through sensitivity analyses, other potential confounding factors may have affected our findings. Therefore, future metabonomic studies on this topic should endeavor to recruit larger patient populations and analyze additional potential confounding factors to enable a more robust study of the phenomenon.

In conclusion, through comparative profiling of the metabolite composition of primary HCC tumors from ALD, HBV-infected, and HCV-infected cirrhotic patients, we found that the key differential metabolites from these three HCC tumor types were significantly different. Moreover, through metabonomic pathway analyses, we determined that these three HCC tumor types displayed significant differences in their affected metabolic pathways. This is the first study to demonstrate that the metabolic phenotypes of primary HCC tumors vary significantly across ALD, HBV-infected, and HCV-infected cirrhotic patients. Further investigation on the differentially dysregulated pathways in ALD, HBV-infected, and HCV-infected HCC tumors should provide valuable insights for tailored chemotherapeutic approaches.

## MATERIALS AND METHODS

### Recruitment of study participants

This study was approved by the Ethics Committees of the Second Affiliated Hospital of Chongqing Medical University (Chongqing, China), the Southwest Hospital of Chongqing (Chongqing, China), and the West China Hospital of Sichuan University (Chengdu, China). All participants provided written informed consent prior to recruitment. HCC candidates at all three hospitals were consecutively selected for initial diagnosis by contrast-enhanced ultrasound and contrast-enhanced computer-aided tomography scanning. Only candidates that received a positive HCC diagnosis with a sT1 classification (a single HCC tumor without vascular invasion and no metastasis) and possessing a cirrhotic liver were included. All candidates were then screened for all major hepatotropic viruses (i.e., hepatitis A, hepatitis B, hepatitis C, hepatitis D, and hepatitis E), schistosomiasis, autoimmune hepatitis, and genetic metabolic liver disease. Candidates with hepatitis A-positive status, schistosomiasis-positive status, autoimmune hepatitis, or genetic metabolic liver disease were excluded. Candidates (i) under the age of 18, (ii) those holding a previous diagnosis of HCC, (iii) those undergoing radiotherapy, chemotherapy, or antiviral therapy, or (iv) those with non-cirrhotic livers were also excluded.

After application of the foregoing inclusion and exclusion criteria, a total of 60 adult HCC patients were finally recruited into this study and segregated into three groups of 20 patients each. The ALD group consisted of 20 first-time cirrhotic HCC patients with ALD and no previous history of HBV or HCV infection (i.e., ALD+, HBV-, HCV-). The HBV group consisted of 20 first-time cirrhotic HCC patients with chronic HBV (defined as IgG anti-HBc+ and HBsAg+ for at least six months) but negative HCV status and no previous history of ALD or alcohol abuse (i.e., HBV+, HCV-, ALD-, no alcohol abuse). The HCV group consisted of 20 first-time cirrhotic HCC patients with chronic HCV (defined as anti-HCV Ab+ and HCV RNA+ for at least six months) but negative HBV status and no previous history of ALD or alcohol abuse (i.e., HCV+, HBV-, ALD-, no alcohol abuse). After recruitment, all patients’ dietary intake were kept uniform to reduce the effects of diet on metabolic profiling.

### Specimen collection

Through liver surgery under general anesthesia, liver tissue specimens from the central area of the primary HCC tumor were collected from all participants. Each specimen was washed in ice-cold normal saline, quickly dried using neutral filter paper, placed on liquid nitrogen, and then stored at-80°C for later metabonomic analysis.

For each patient, all relevant demographic and clinical characteristics were collected, including: patient age (years), patient sex (male or female), clinical risk factors(i.e., diabetes status, BMI, smoking status, level of alcohol consumption (g/week)), relevant serum biochemical markers (i.e., ALT, AST, alkaline phosphatase, total bilirubin, direct bilirubin, and albumin), recommended HCC scoring scales from MD Anderson Cancer Center’s current practice algorithm (http://www.mdanderson.org/) (i.e., ECOG performance status score, CLIP score, and Child-Pugh score), and relevant tumor information (i.e., size of tumor (cm) and pathological grade of tumor cells (G1-G4)).

### HRMAS NMR spectroscopy

The protocol was performed as previously described by Solinas et al. with minor modifications^7^. Briefly, ^1^H HRMAS NMR was performed at 277 K using a Bruker AVANCE II 600 MHz spectrometer. Each specimen was washed in D_2_O to remove any blood and/or debris. Each specimen was then placed into the HRMAS rotor with 5 μl phosphate buffered saline (PBS, pH 7.2) and 10% D_2_O and 3-(trimethylsilyl)propionic-2,2,3,3-d4 acid sodium salt (TSP, 0.01%) for referencing. The specimens were spun at 4000 Hz, and a water-suppressed spin-echo Carr-Purcell-Meiboom-Gill (CPMG) pulse sequence was acquired with a 2.5-s water pre-saturation during relaxation delay, a 1-ms echo time (τ), and a 40-ms total spin-spin relaxation delay (2nτ). Spectra were acquired with 128 scans into 32 K data points with a spectral width of 8-KHz. The spectral sections between 4.5 and 5.5 ppm (residual H_2_O resonance) were excluded. Two-dimensional (2D) 1H-13C-HSQC spectra were recorded with spectral widths of 9920 Hz in the F2 dimension and 33936 Hz in the F1 dimension, 1400 × 512 data points, and 48 scans per increment.

Bruker TOPSPIN 3.2 was used to process the resulting data. Metabolites were identified with the BBIOREFCODE 2.0.2 database (Bruker Bio-Spin GmbH Rheinstetten, Germany), HMDB database, and relevant literature. Assignments of the significant metabolites were validated by 2D TOCSY and 2D 1H-13C-HSQC spectral analysis.

### Metabonomic data analysis

As previously described by Solinas et al. with minor modifications^7^, metabonomic data analysis was applied to perform three comparisons: ALD group versus HBV group, ALD group versus HCV group, and HBV group versus HCV group. Following exclusion of the distorted regions from water suppression (4.5-5.5 ppm), the resulting 9.5-5.5 ppm and 4.5-0.54 ppm spectral regions were normalized to 100. With Analysis of MIXtures (AMIX) (Bruker GmbH, Karlsruhe, Germany), the 199 0.04-ppm consecutively integrated segments were created in each spectrum. The data was transferred into an Excel spreadsheet for purposes of labeling, and SIMCA-P version 13.0 (Umetrics AB, Umeå, Sweden) was used to statistically analyze the data. All data were mean-centered.

Orthogonal partial least square discriminant analysis (OPLS-DA) was used to perform the supervised analysis with the number of the orthogonal components calculated by SIMCA-P’s ‘autofit’ routine. The PLS-DA models were validated by 200× permutation testing to prevent model overfitting. Each model’s predictive ability was assessed by leave-one-out analysis. Non-parametric U Mann-Whitney analysis was used to compare peak integrals.

In order to determine the effects of diabetes, high BMI, and smoking status on the metabonomic findings, the foregoing analysis procedure was re-performed on three sensitivity subgroups as follows: (i) a diabetes sensitivity analysis was performed after removing diabetic patients from the analysis, (ii) a BMI sensitivity analysis was performed after removing overweight patients (BMI≥25) from the analysis, and (iii) a smoking sensitivity analysis was performed after removing smokers from the analysis.

### Metabonomic pathway analysis

For the metabonomic pathway analysis, the levels of the key metabolites (both before and after applying the sensitivity analysis) were uploaded into MetaboAnalyst 3.0 -- an online tool that integrates pathway enrichment analysis with pathway topology analysis in order to identify the most relevant affected pathways [13]. The following settings were applied for the pathway analysis: sample normalization (none), data scaling (none), species (*Homo sapiens*), pathway enrichment analysis algorithm (Global Test), and pathway topology analysis algorithm (Relative-betweeness Centrality).

## SUPPLEMENTARY FIGURES AND TABLES


