# Supplementary material for: Differential metabonomic profiles of primary hepatocellular carcinoma tumors from alcoholic liver disease, HBV-infected, and HCV-infected cirrhotic patients
Source: Oncotarget. 2017 Jun 7;8(32):53313–25. doi: 10.18632/oncotarget.18397 (PMC5581112; doi:10.18632/oncotarget.18397)
Supplement: Supplementary file 1 [file oncotarget-08-53313-s001.pdf]

Differential metabonomic profiles of primary hepatocellular carcinoma tumors from alcoholic liver disease, HBV-infected, and HCV-infected cirrhotic patients

SUPPLEMENTARY MATERIALS

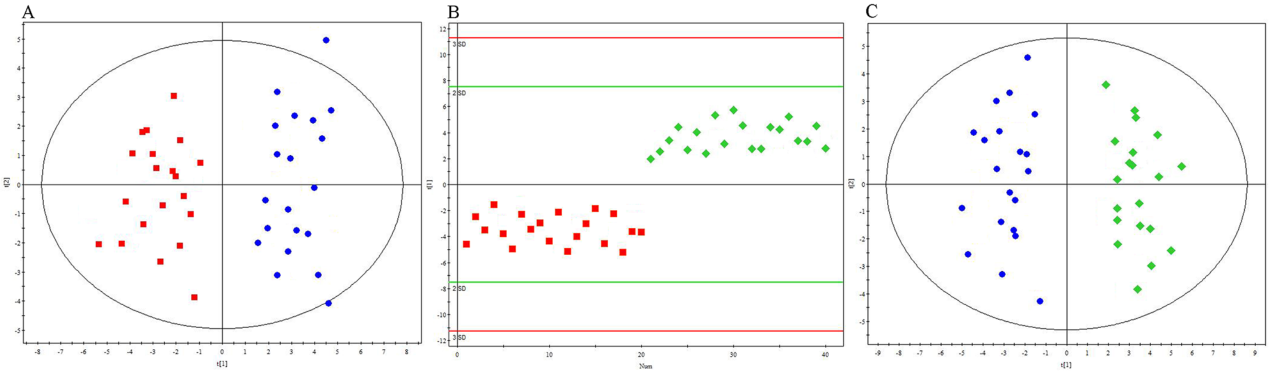

Supplementary Figure 1: Non-Supervised Principal Component Analysis (PCA). (A) Non-supervised PCA score plot showing a clear discrimination between primary HCC tumors from ALD patients (red squares) and HBV-infected patients (blue circles). $R^2X[1] = 0.285$, $R^2X[2] = 0.107$. **(B)** Non-supervised PCA score plot showing a clear discrimination between primary HCC tumors from ALD patients (red squares) and HCV-infected patients (green diamonds). $R^2X[1] = 0.411$. **(C)** Non-supervised PCA score plot showing a clear discrimination between primary HCC tumors from HBV-infected patients (blue circles) and HCV-infected patients (green diamonds). $R^2X[1] = 0.325$, $R^2X[2] = 0.124$.

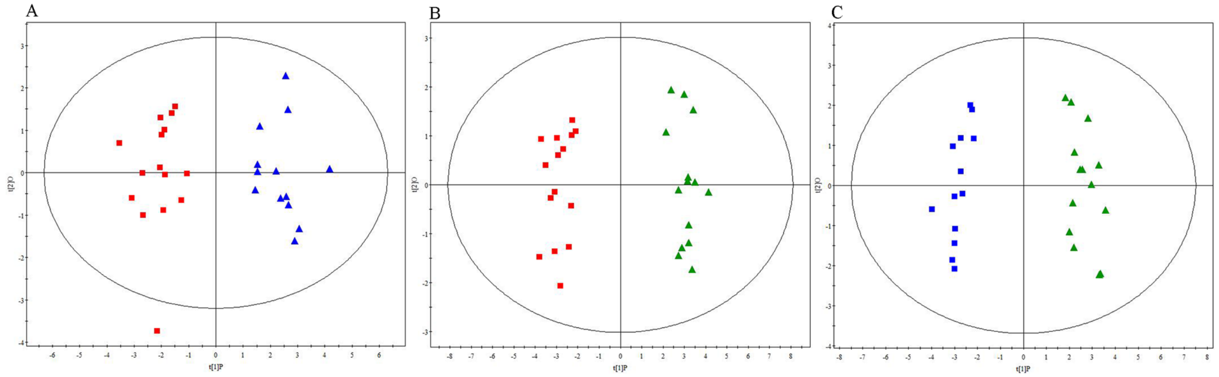

Supplementary Figure 2: Diabetes Sensitivity Analysis. (A) After excluding diabetics, OPLS-DA score plots showing a clear discrimination between primary HCC tumors from ALD patients (red squares) and HBV-infected patients (blue circles). R^2=0.913, Q^2=0.766. **(B)** After excluding diabetics, OPLS-DA score plots showing a clear discrimination between primary HCC tumors from ALD patients (red squares) and HCV-infected patients (green diamonds). R^2=0.972, Q^2=0.926. **(C)** After excluding diabetics, OPLS-DA score plots showing a clear discrimination between primary HCC tumors from HBV-infected patients (blue circles) and HCV-infected patients (green diamonds). R^2=0.966, Q^2=0.929.

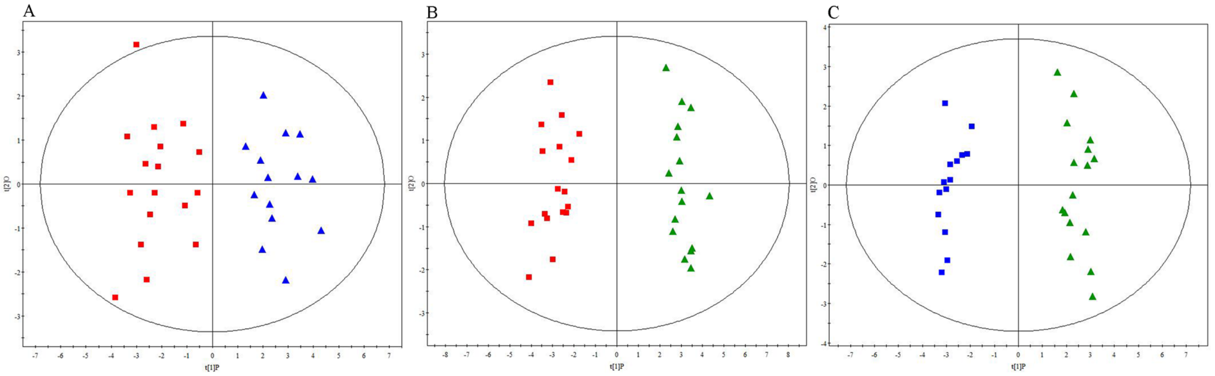

Supplementary Figure 3: BMI Sensitivity Analysis. (A) After excluding overweight individuals, OPLS-DA score plots showing a clear discrimination between primary HCC tumors from ALD patients (red squares) and HBV-infected patients (blue circles). R^2=0.928, Q^2=0.787. **(B)** After excluding overweight individuals, OPLS-DA score plots showing a clear discrimination between primary HCC tumors from ALD patients (red squares) and HCV-infected patients (green diamonds). R^2=0.905, Q^2=0.827. **(C)** After excluding overweight individuals, OPLS-DA score plots showing a clear discrimination between primary HCC tumors from HBV-infected patients (blue circles) and HCV-infected patients (green diamonds). R^2=0.971, Q^2=0.929.

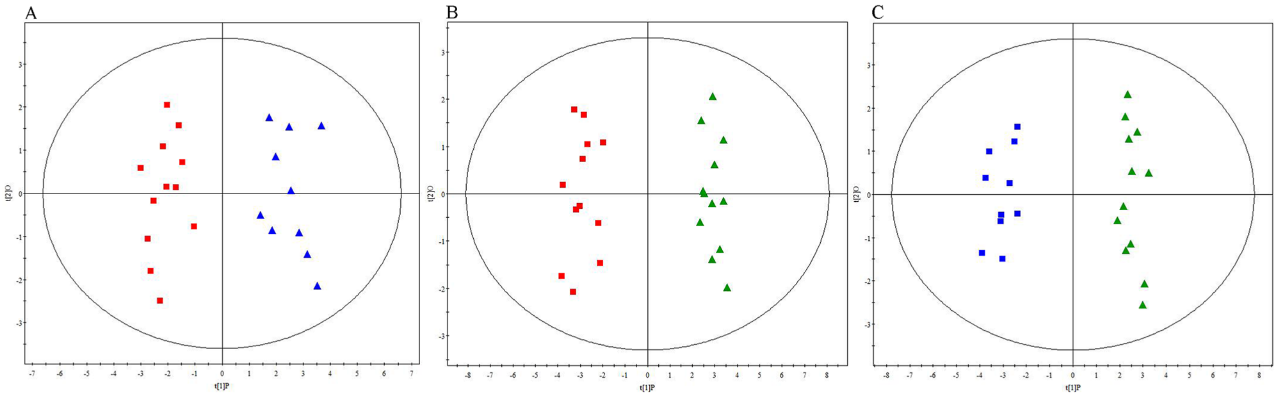

Supplementary Figure 4: Smoking Sensitivity Analysis. (A) After excluding smokers, OPLS-DA score plots showing a clear discrimination between primary HCC tumors from ALD patients (red squares) and HBV-infected patients (blue circles). R^2=0.866, Q^2=0.697. **(B)** After excluding smokers, OPLS-DA score plots showing a clear discrimination between primary HCC tumors from ALD patients (red squares) and HCV-infected patients (green diamonds). R^2=0.972, Q^2=0.913. **(C)** After excluding smokers, OPLS-DA score plots showing a clear discrimination between primary HCC tumors from HBV-infected patients (blue circles) and HCV-infected patients (green diamonds). R^2=0.974, Q^2=0.932.

Supplementary Table 1: Key Metabolites Responsible for Discriminating between Primary HCC Tumors from ALD Patients and HBV-Infected Patients

Chemical shift/ppm (multiplicity)[a]	Metabolites	r[b]	P-value[c]
3.54(s)	Glycine	-0.501	3.36E-5
3.13(d), 3.81(d)	Ethanolamine	-0.478	1.34E-7
3.55(m), 3.64(m)	Glycerol	-0.501	5.02E-7
6.85(d), 7.15(d)	*p*-hydroxyphenylacetate	-0.454	4.72E-4
2.27(s), 3.43(s)	Acetoacetate	-0.604	5.08E-8

HCC: hepatocellular carcinoma; ALD: alcoholic liver disease; HBV: hepatitis B virus.
[a]Multiplicity: s: singlet; d: doublet; t: triplet; q: quartet; dd: doublet of doublets; m: multiplet.
[b]The correlation coefficient (r) was obtained from OPLS-DA with a threshold of 0.444. Positive values indicate higher levels in HCC tumors from HBV-infected patients, while negative values indicate lower levels in HCC tumors from HBV-infected patients.
[c]P-values were derived from non-parametric Mann-Whitney U analysis.

Supplementary Table 2: Key Metabolites Responsible for Discriminating between Primary HCC Tumors from ALD Patients and HCV-Infected Patients

Chemical shift/ppm (multiplicity)[a]	Metabolites	r[b]	P-value[c]
3.25(s)	Trimethylamine N-oxide	0.486	1.43E-3
3.02(s), 3.92(s)	Creatine	-0.582	1.45E-11
3.54(s)	Glycine	-0.541	5.41E-9
1.46(d), 3.76(q)	Alanine	-0.712	1.45E-11
7.29(m), 7.36(m)	Phenylacetate	-0.631	4.35E-10
8.17(s), 8.20(s)	Hypoxanthine	-0.661	1.45E-11
2.88(s)	Trimethylamine	0.585	4.19E-4
2.27(s), 3.43(s)	Acetoacetate	-0.797	1.45E-11

HCC: hepatocellular carcinoma; ALD: alcoholic liver disease; HCV: hepatitis C virus; NADP: nicotinamide adenine dinucleotide phosphate.
[a]Multiplicity: s: singlet; d: doublet; t: triplet; q: quartet; dd: doublet of doublets; m: multiplet.
[b]The correlation coefficient (r) was obtained from OPLS-DA with a threshold of 0.444. Positive values indicate higher levels in HCC tumors from HCV-infected patients, while negative values indicate lower levels in HCC tumors from HCV-infected patients.
[c]P-values were derived from non-parametric Mann-Whitney U analysis.

Supplementary Table 3: Key Metabolites Responsible for Discriminating between Primary HCC Tumors from HBV-Infected Patients and HCV-Infected Patients

Chemical shift/ppm (multiplicity)[a]	Metabolites	*r*[b]	*P*-value[c]
3.27(s)	Trimethylamine N-oxide	0.562	9.33E-4
3.02(s), 3.92(s)	Creatine	-0.627	1.76E-8
1.46(d), 3.76(q)	Alanine	-0.765	1.45E-11
7.29(m), 7.36(m)	Phenylacetate	-0.864	1.45E-11
8.21(s), 8.49(s)	NADP	0.918	1.29E-3
8.17(s), 8.20(s)	Hypoxanthine	-0.554	8.34E-8
6.85(d), 7.15(d)	*p*-hydroxyphenylacetate	0.999	1.77E-3
2.27(s), 3.43(s)	Acetoacetate	-0.622	7.37E-9

HCC: hepatocellular carcinoma; ALD: alcoholic liver disease; HCV: hepatitis C virus; NADP: nicotinamide adenine dinucleotide phosphate.

[a]Multiplicity: s: singlet; d: doublet; t: triplet; q: quartet; dd: doublet of doublets; m: multiplet.

[b]The correlation coefficient (*r*) was obtained from OPLS-DA with a threshold of 0.444. Positive values indicate higher levels in HCC tumors from HCV-infected patients, while negative values indicate lower levels in HCC tumors from HCV-infected patients.

[c]*P*-values were derived from non-parametric Mann-Whitney U analysis.

Supplementary Table 4: Diabetes Sensitivity Analysis: Key Metabolites Responsible for Discriminating between Primary HCC Tumors from ALD Patients and HBV-Infected Patients Excluding Diabetic Individuals

Chemical shift/ppm (multiplicity)[a]	Metabolites	*r*[b]	*P*-value[c]
6.85(d), 7.15(d)	*p*-hydroxyphenylacetate	-0.506	4.39E-3
3.27(t), 3.43(t)	Taurine	-0.457	1.28E-2
8.46(s)	Formate	0.751	2.43E-2
2.27(s), 3.43(s)	Acetoacetate	-0.568	1.99E-5
3.55(m), 3.64(m)	Glycerol	-0.480	6.38E-5

HCC: hepatocellular carcinoma; ALD: alcoholic liver disease; HBV: hepatitis B virus.

[a]Multiplicity: s: singlet; d: doublet; t: triplet; q: quartet; dd: doublet of doublets; m: multiplet.

[b]The correlation coefficient (*r*) was obtained from OPLS-DA with a threshold of 0.444. Positive values indicate higher levels in HCC tumors from HBV-infected patients, while negative values indicate lower levels in HCC tumors from HBV-infected patients.

[c]*P*-values were derived from non-parametric Mann-Whitney U analysis.

Supplementary Table 5: Diabetes Sensitivity Analysis: Key Metabolites Responsible for Discriminating between Primary HCC Tumors from ALD Patients and HCV-Infected Patients Excluding Diabetic Individuals

Chemical shift/ppm (multiplicity)[a]	Metabolites	*r*[b]	*P*-value[c]
3.02(s), 3.92(s)	Creatine	-0.726	2.57E-8
1.92(s)	Acetate	-0.575	2.50E-5
2.41(s)	Succinate	-0.524	9.61E-5
7.29(m), 7.36(m)	Phenylacetate	-0.521	3.09E-7
2.88(s)	Trimethylamine	0.680	9.62E-6
3.54(s)	Glycine	-0.544	5.03E-6
2.27(s), 3.43(s)	Acetoacetate	-0.823	2.57E-8
3.25(s)	Trimethylamine N-oxide	0.547	2.67E-3
1.46(d), 3.76(q)	Alanine	-0.741	2.57E-8
3.55(m), 3.64(m)	Glycerol	-0.541	6.81E-5

HCC: hepatocellular carcinoma; ALD: alcoholic liver disease; HCV: hepatitis C virus; NADP: nicotinamide adenine dinucleotide phosphate.
[a]Multiplicity: s: singlet; d: doublet; t: triplet; q: quartet; dd: doublet of doublets; m: multiplet.
[b]The correlation coefficient (*r*) was obtained from OPLS-DA with a threshold of 0.444. Positive values indicate higher levels in HCC tumors from HCV-infected patients, while negative values indicate lower levels in HCC tumors from HCV-infected patients.
[c]*P*-values were derived from non-parametric Mann-Whitney U analysis.

Supplementary Table 6: Diabetes Sensitivity Analysis: Key Metabolites Responsible for Discriminating between Primary HCC Tumors from HBV-Infected Patients and HCV-Infected Patients Excluding Diabetic Individuals

Chemical shift/ppm (multiplicity)[a]	Metabolites	*r*[b]	*P*-value[c]
3.02(s), 3.92(s)	Creatine	-0.765	9.67E-6
6.85(d), 7.15(d)	*p*-hydroxyphenylacetate	0.999	4.81E-2
7.29(m), 7.36(m)	Phenylacetate	-0.889	9.97E-8
2.88(s)	Trimethylamine	0.522	5.05E-5
8.21(s), 8.49(s)	NADP	0.954	2.27E-3
8.17(s), 8.20(s)	Hypoxanthine	-0.595	9.02E-5
2.27(s), 3.43(s)	Acetoacetate	-0.698	4.48E-6
3.25(s)	Trimethylamine N-oxide	0.444	5.59E-3
1.46(d), 3.76(q)	Alanine	-0.738	9.97E-8

HCC: hepatocellular carcinoma; ALD: alcoholic liver disease; HCV: hepatitis C virus; NADP: nicotinamide adenine dinucleotide phosphate.
[a]Multiplicity: s: singlet; d: doublet; t: triplet; q: quartet; dd: doublet of doublets; m: multiplet.
[b]The correlation coefficient (*r*) was obtained from OPLS-DA with a threshold of 0.444. Positive values indicate higher levels in HCC tumors from HCV-infected patients, while negative values indicate lower levels in HCC tumors from HCV-infected patients.
[c]*P*-values were derived from non-parametric Mann-Whitney U analysis.

Supplementary Table 7: BMI Sensitivity Analysis: Key Metabolites Responsible for Discriminating between Primary HCC Tumors from ALD Patients and HBV-Infected Patients Excluding Overweight Individuals (BMI>=25)

Chemical shift/ppm (multiplicity)[a]	Metabolites	*r*[b]	*P*-value[c]
6.85(d), 7.15(d)	*p*-hydroxyphenylacetate	-0.607	1.17E-4
3.54(s)	Glycine	-0.446	4.36E-4
8.17(s), 8.20(s)	Hypoxanthine	-0.494	2.60E-4
3.27(t), 3.43(t)	Taurine	-0.500	1.30E-3
2.27(s), 3.43(s)	Acetoacetate	-0.594	1.26E-5
1.47(m), 3.04(t)	Lysine	-0.455	5.00E-6
3.55(m), 3.64(m)	Glycerol	-0.559	3.07E-5

HCC: hepatocellular carcinoma; ALD: alcoholic liver disease; HBV: hepatitis B virus.
[a]Multiplicity: s: singlet; d: doublet; t: triplet; q: quartet; dd: doublet of doublets; m: multiplet.
[b]The correlation coefficient (*r*) was obtained from OPLS-DA with a threshold of 0.444. Positive values indicate higher levels in HCC tumors from HBV-infected patients, while negative values indicate lower levels in HCC tumors from HBV-infected patients.
[c]*P*-values were derived from non-parametric Mann-Whitney U analysis.

Supplementary Table 8: BMI Sensitivity Analysis: Key Metabolites Responsible for Discriminating between Primary HCC Tumors from ALD Patients and HCV-Infected Patients Excluding Overweight Individuals (BMI>=25)

Chemical shift/ppm (multiplicity)[a]	Metabolites	*r*[b]	*P*-value[c]
3.02(s), 3.92(s)	Creatine	-0.647	9.63E-7
1.92(s)	Acetate	-0.784	1.38E-6
7.29(m), 7.36(m)	Phenylacetate	-0.709	9.63E-7
2.88(s)	Trimethylamine	0.637	1.04E-3
8.17(s), 8.20(s)	Hypoxanthine	-0.615	9.63E-7
2.27(s), 3.43(s)	Acetoacetate	-0.805	9.63E-7
3.25(s)	Trimethylamine N-oxide	0.671	3.95E-3
1.46(d), 3.76(q)	Alanine	-0.662	9.63E-7

HCC: hepatocellular carcinoma; ALD: alcoholic liver disease; HCV: hepatitis C virus; NADP: nicotinamide adenine dinucleotide phosphate.
[a]Multiplicity: s: singlet; d: doublet; t: triplet; q: quartet; dd: doublet of doublets; m: multiplet.
[b]The correlation coefficient (*r*) was obtained from OPLS-DA with a threshold of 0.444. Positive values indicate higher levels in HCC tumors from HCV-infected patients, while negative values indicate lower levels in HCC tumors from HCV-infected patients.
[c]*P*-values were derived from non-parametric Mann-Whitney U analysis.

Supplementary Table 9: BMI Sensitivity Analysis: Key Metabolites Responsible for Discriminating between Primary HCC Tumors from HBV-Infected Patients and HCV-Infected Patients Excluding Overweight Individuals (BMI>=25)

Chemical shift/ppm (multiplicity)[a]	Metabolites	*r*[b]	*P*-value[c]
3.02(s), 3.92(s)	Creatine	-0.681	1.05E-6
6.85(d), 7.15(d)	*p*-hydroxyphenylacetate	0.999	5.34E-3
2.54(d), 2.69(d)	Citrate	0.850	1.82E-3
8.21(s), 8.49(s)	NADP	0.866	2.41E-3
8.17(s), 8.20(s)	Hypoxanthine	-0.586	1.55E-4
2.27(s), 3.43(s)	Acetoacetate	-0.494	3.86E-5
3.25(s)	Trimethylamine N-oxide	0.484	1.12E-2
1.46(d), 3.76(q)	Alanine	-0.729	3.23E-6
1.47(m), 3.04(t)	Lysine	-0.476	4.62E-5

HCC: hepatocellular carcinoma; ALD: alcoholic liver disease; HCV: hepatitis C virus; NADP: nicotinamide adenine dinucleotide phosphate.
[a]Multiplicity: s: singlet; d: doublet; t: triplet; q: quartet; dd: doublet of doublets; m: multiplet.
[b]The correlation coefficient (*r*) was obtained from OPLS-DA with a threshold of 0.444. Positive values indicate higher levels in HCC tumors from HCV-infected patients, while negative values indicate lower levels in HCC tumors from HCV-infected patients.
[c]*P*-values were derived from non-parametric Mann-Whitney U analysis.

Supplementary Table 10: Smoking Sensitivity Analysis: Key Metabolites Responsible for Discriminating between Primary HCC Tumors from ALD Patients and HBV-Infected Patients Excluding Smokers

Chemical shift/ppm (multiplicity)[a]	Metabolites	*r*[b]	*P*-value[c]
6.85(d), 7.15(d)	*p*-hydroxyphenylacetate	-0.459	4.79E-2
8.21(s), 8.49(s)	NADP	-0.477	1.94E-3
3.54(s)	Glycine	-0.548	9.77E-4
3.13(d), 3.81(d)	Ethanolamine	-0.650	7.61E-5
2.27(s), 3.43(s)	Acetoacetate	-0.777	3.69E-4
3.55(m), 3.64(m)	Glycerol	-0.688	1.71E-4

HCC: hepatocellular carcinoma; ALD: alcoholic liver disease; HBV: hepatitis B virus.
[a]Multiplicity: s: singlet; d: doublet; t: triplet; q: quartet; dd: doublet of doublets; m: multiplet.
[b]The correlation coefficient (*r*) was obtained from OPLS-DA with a threshold of 0.444. Positive values indicate higher levels in HCC tumors from HBV-infected patients, while negative values indicate lower levels in HCC tumors from HBV-infected patients.
[c]*P*-values were derived from non-parametric Mann-Whitney U analysis.

Supplementary Table 11: Smoking Sensitivity Analysis: Key Metabolites Responsible for Discriminating between Primary HCC Tumors from ALD Patients and HCV-Infected Patients Excluding Smokers

Chemical shift/ppm (multiplicity)[a]	Metabolites	*r*[b]	*P*-value[c]
3.02(s), 3.92(s)	Creatine	-0.482	3.22E-5
7.29(m), 7.36(m)	Phenylacetate	-0.587	8.63E-5
3.54(s)	Glycine	-0.661	3.22E-5
8.17(s), 8.20(s)	Hypoxanthine	-0.608	3.22E-5
3.27(t), 3.43(t)	Taurine	-0.468	3.22E-5
2.27(s), 3.43(s)	Acetoacetate	-0.865	3.22E-5
3.25(s)	Trimethylamine N-oxide	0.486	1.82E-3
1.46(d), 3.76(q)	Alanine	-0.829	3.22E-5

HCC: hepatocellular carcinoma; ALD: alcoholic liver disease; HCV: hepatitis C virus; NADP: nicotinamide adenine dinucleotide phosphate.
[a]Multiplicity: s: singlet; d: doublet; t: triplet; q: quartet; dd: doublet of doublets; m: multiplet.
[b]The correlation coefficient (*r*) was obtained from OPLS-DA with a threshold of 0.444. Positive values indicate higher levels in HCC tumors from HCV-infected patients, while negative values indicate lower levels in HCC tumors from HCV-infected patients.
[c]*P*-values were derived from non-parametric Mann-Whitney U analysis.

Supplementary Table 12: Smoking Sensitivity Analysis: Key Metabolites Responsible for Discriminating between Primary HCC Tumors from HBV-Infected Patients and HCV-Infected Patients Excluding Smokers

Chemical shift/ppm (multiplicity)[a]	Metabolites	*r*[b]	*P*-value[c]
6.85(d), 7.15(d)	*p*-hydroxyphenylacetate	0.999	1.76E-2
7.29(m), 7.36(m)	Phenylacetate	-0.788	7.61E-5
8.21(s), 8.49(s)	NADP	0.915	8.23E-3
8.17(s), 8.20(s)	Hypoxanthine	-0.642	1.70E-4
3.20(s), 4.07(s)	Choline	-0.465	7.61E-5
2.27(s), 3.43(s)	Acetoacetate	-0.568	3.69E-4
3.25(s)	Trimethylamine N-oxide	0.658	1.94E-3
1.46(d), 3.76(q)	Alanine	-0.671	7.61E-5
1.47(m), 3.04(t)	Lysine	-0.528	1.31E-4

HCC: hepatocellular carcinoma; ALD: alcoholic liver disease; HCV: hepatitis C virus; NADP: nicotinamide adenine dinucleotide phosphate.
[a]Multiplicity: s: singlet; d: doublet; t: triplet; q: quartet; dd: doublet of doublets; m: multiplet.
[b]The correlation coefficient (*r*) was obtained from OPLS-DA with a threshold of 0.444. Positive values indicate higher levels in HCC tumors from HCV-infected patients, while negative values indicate lower levels in HCC tumors from HCV-infected patients.
[c]*P*-values were derived from non-parametric Mann-Whitney U analysis.
